# Neural Marker Expression in Adipose-Derived Stem Cells Grown in PEG-Based 3D Matrix Is Enhanced in the Presence of B27 and CultureOne Supplements

**DOI:** 10.3390/ijms242216269

**Published:** 2023-11-13

**Authors:** Neus Gomila Pelegri, Aleksandra M. Stanczak, Amy L. Bottomley, Max L. Cummins, Bruce K. Milthorpe, Catherine A. Gorrie, Matthew P. Padula, Jerran Santos

**Affiliations:** 1 Advanced Tissue Engineering and Stem Cell Biology Group, School of Life Sciences, University of Technology Sydney, Ultimo, NSW 2007, Australia; neus.gomilapelegri@uts.edu.au (N.G.P.); bruce.milthorpe@uts.edu.au (B.K.M.); 2 Neural Injury Research Unit, School of Life Sciences, University of Technology Sydney, Ultimo, NSW 2007, Australia; catherine.gorrie@uts.edu.au; 3 School of Life Sciences, University of Technology Sydney, Ultimo, NSW 2007, Australia; aleksandra.m.stanczak@student.uts.edu.au (A.M.S.); matthew.padula@uts.edu.au (M.P.P.); 4 Microbial Imaging Facility, University of Technology Sydney, Ultimo, NSW 2007, Australia; amy.bottomley@uts.edu.au; 5 Australian Institute for Microbiology and Infection, University of Technology Sydney, Ultimo, NSW 2007, Australia; max.cummins@uts.edu.au; 6 The Australian Centre for Genomic Epidemiological Microbiology, University of Technology Sydney, Ultimo, NSW 2007, Australia

**Keywords:** tissue engineering, bioprinting, neural differentiation, proteomics, 3D tissue culture, immunocytochemistry, polyethylene glycol, neuroregeneration, neural development, regenerative medicine

## Abstract

Adipose-derived stem cells (ADSCs) have incredible potential as an avenue to better understand and treat neurological disorders. While they have been successfully differentiated into neural stem cells and neurons, most such protocols involve 2D environments, which are not representative of in vivo physiology. In this study, human ADSCs were cultured in 1.1 kPa polyethylene-glycol 3D hydrogels for 10 days with B27, CultureOne (C1), and N2 neural supplements to examine the neural differentiation potential of ADSCs using both chemical and mechanical cues. Following treatment, cell viability, proliferation, morphology, and proteome changes were assessed. Results showed that cell viability was maintained during treatments, and while cells continued to proliferate over time, proliferation slowed down. Morphological changes between 3D untreated cells and treated cells were not observed. However, they were observed among 2D treatments, which exhibited cellular elongation and co-alignment. Proteome analysis showed changes consistent with early neural differentiation for B27 and C1 but not N2. No significant changes were detected using immunocytochemistry, potentially indicating a greater differentiation period was required. In conclusion, treatment of 3D-cultured ADSCs in PEG-based hydrogels with B27 and C1 further enhances neural marker expression, however, this was not observed using supplementation with N2.

## 1. Introduction

Adipose-derived stem cells (ADSCs) have been at the forefront of regenerative medicine, given their potential to differentiate into adipogenic, chondrogenic, osteogenic, myogenic, and neurogenic-like cell lineages [1,2,3,4]. These qualities make them suitable for many applications, and in the context of neuroregeneration, ADSCs are a desirable source of cells due to their neurogenic differentiation potential [2,3,4] and their availability [5,6]. Autologous ADSCs can be easily obtained in high numbers via subcutaneous adipose liposuction, which is a significantly less invasive procedure when compared to other collection methods like bone marrow aspirates [5,6,7].

ADSCs have been successfully differentiated into neural-like cells using different growth factors and chemicals [8,9,10,11,12,13,14]. Rodent ADSCs have been successfully differentiated into neural stem cells and dopaminergic neurons [8], while human ADSCs (hADSCs) have also shown neurogenic potential and have been successfully differentiated into neurospheres [9], neuron-like cells [9], and dopamine-secreting cells [10,11]. Furthermore, transplantation of ADSCs has shown positive effects in animal models of neurological disorders such as Parkinson’s disease [15], peripheral nerve injury [16], epilepsy [17], and stroke [18].

These findings are promising and show ADSCs’ potential as a tool to understand neural development and find potential treatments for neurological disorders. However, the most common methods of differentiation are usually solely chemically induced, using multistep protocols with many chemical mixtures, and performed in 2D cell culture environments [2,3,8,9,10,11,12,13,14,19]. These monolayer cellular models are not representative of human tissue structure and do not consider any of the crucial 3D interactions that affect stem cells during differentiation [20,21]. Some of these chemical inductions have proven to be transient and reversible when conducted in a 2D environment alone; Ahmadi et al. demonstrated that a neurosphere formation protocol, while slower than chemical inductions, had better cell viability, and the neural differentiation was more stable than in 2D constructs [9].

In recent years, research has shown that cells grown in a 3D environment that mimics their native tissue are more comparable to in vivo behaviour and present distinctive morphological changes compared to their 2D-grown counterparts [20,21,22,23]. In a 3D environment, cells can arrange themselves in more natural conformations. They can aggregate themselves into multiple layers where cell exposure to nutrients and waste products is similar to in vitro conditions [21,22,24]. These arrangements provide insights into drug treatments and cell proliferation rates that are more realistic than those observed in 2D cultures [25]. Furthermore, it has been shown that cells respond to the mechanical stimuli of their environment, such as pressure, and interact with ECM proteins and scaffold composition, mimicking the natural interactions between the cells and their ECM and directing cellular differentiation [26,27,28,29]. Of particular interest, human-derived mesenchymal stem cells (hMSCs) have been shown to differentiate towards a neural lineage when grown in scaffolds resembling a brain stiffness of 1 kPa [28]. Additionally, our previous work showed that hADSCs spontaneously express neural markers when grown in PEG-based hydrogels of 1.1 kPa stiffness [30], further highlighting the importance of growth matrices and cell growth environment when conducting neural differentiation of hADSCs.

This study builds upon our previous work, where we used the readily available media supplements B27, C1, and N2 for neural induction of ADSCs in 2D matrices [31], and on our most recent investigation of the effects of mechanical properties of 3D polyethylene glycol (PEG)-based hydrogels containing RGD and YIGSR peptides to initiate neural differentiation of hADSCs [30]. This work merges both concepts and explores the effects of treatment and matrix combined using cost-effective and standardised supplements together with the reproducibility of commercially available system rastrum bioprinting (Inventia, Sydney, Australia). This is necessary to progress the development of treatments for a range of conditions where neuronal tissue is compromised.

## 2. Results

### 2.1. Cell Proliferation, Viability, and Morphology

#### 2.1.1. Cell Proliferation and Viability

**Figure 1:**
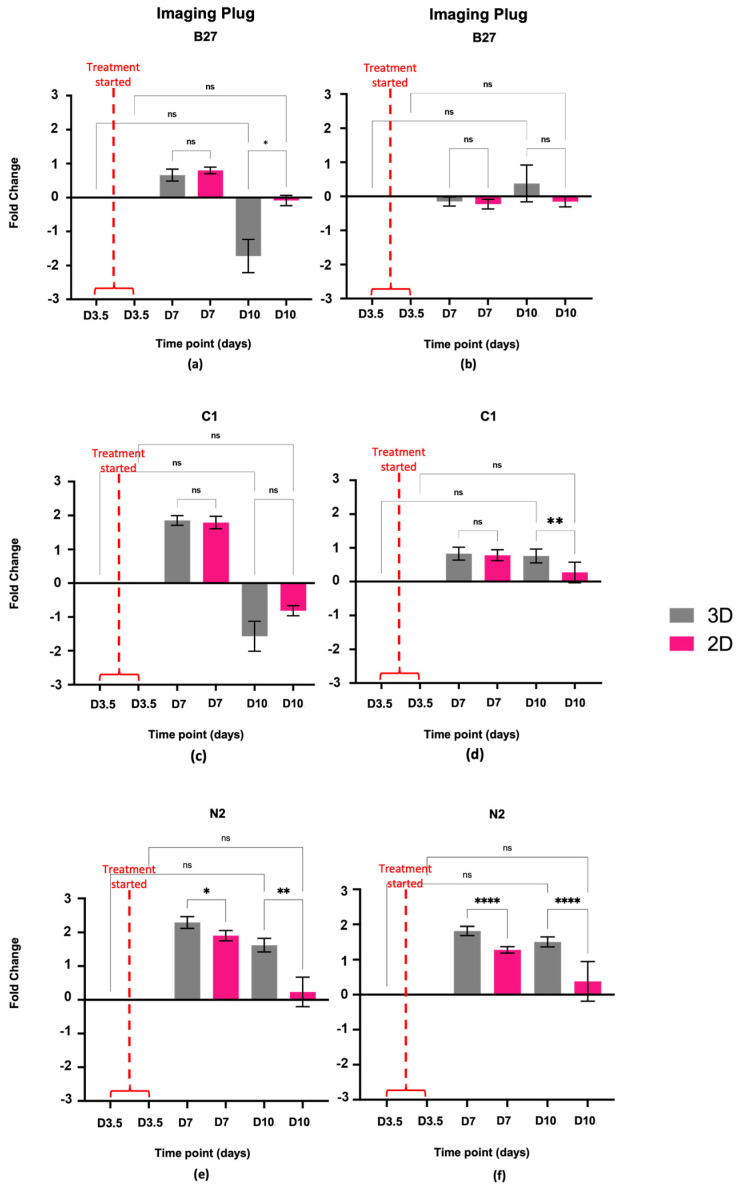
Alamar blue cell viability. Fold change activity measured over treatment course using Alamar blue assay. Fold change is relative to day 3.5 prior to cell treatment, marked by the red line. A Log2 scale has been used where the initial measurement obtained on day 3.5 equals zero, and the increase or decrease in measured parameters falls on the respective side of the x-axis. (**a**) Imaging plug treated with B27, (**b**) large plug treated with B27, (**c**) imaging plug treated with C1, (**d**) large plug treated with C1, (**e**) imaging plug treated with N2, (**f**) large plug treated with N2. No statistical significance *p* > 0.05; statistical significance *, *p* ≤ 0.05; statistical significance **, *p* ≤ 0.01; statistical significance ****, *p* ≤ 0.0001; ns: not significant.

Cell treatment began on day 3.5, 84 h after the cells were plated. The Alamar blue measurement on day 3.5 was the last measurement before cells started treatment and therefore serves as a control. Increases or decreases in fold change and hence cell metabolic activity were measured from that time point. An increase in fold change is indicative of cells showing an increase in cell metabolic activity, while a decrease in fold change is indicative of a decrease in metabolic activity (Figure 1).

Alamar blue results for cells treated with B27 show very little change in metabolic activity for both 3D and 2D samples (Figure 1a,b). In the imaging plug, metabolic activity decreased in the 3D samples compared to the 2D samples at D10 (Figure 1a) but no statistically significant changes were detected in the 3D plug (Figure 1b).

For the cells treated with C1, Alamar blue results show that metabolic activity decreased in the imaging plug for both 3D and 2D samples from D3 to D10 (Figure 1c), however, no statistical significance was detected. In the large plug, metabolic activity increased in both the 3D and the 2D samples compared to the control, with 2D samples showing significantly lower metabolic activity at D10 compared to the 3D samples (Figure 1d) (*p* ≤ 0.05; statistical significance **).

Alamar blue results indicate that N2 treatment causes metabolic activity to increase at the beginning of treatment and then decrease by day 10 in the imaging and large plugs for both 3D and 2D samples (Figure 1e,f), with 3D samples having overall higher metabolic activity compared to 2D samples (*, *p* ≤ 0.05; statistical significance **, *p* ≤ 0.01; statistical significance for imaging plug, and statistical significance ****, *p* ≤ 0.0001).

**Figure 2:**
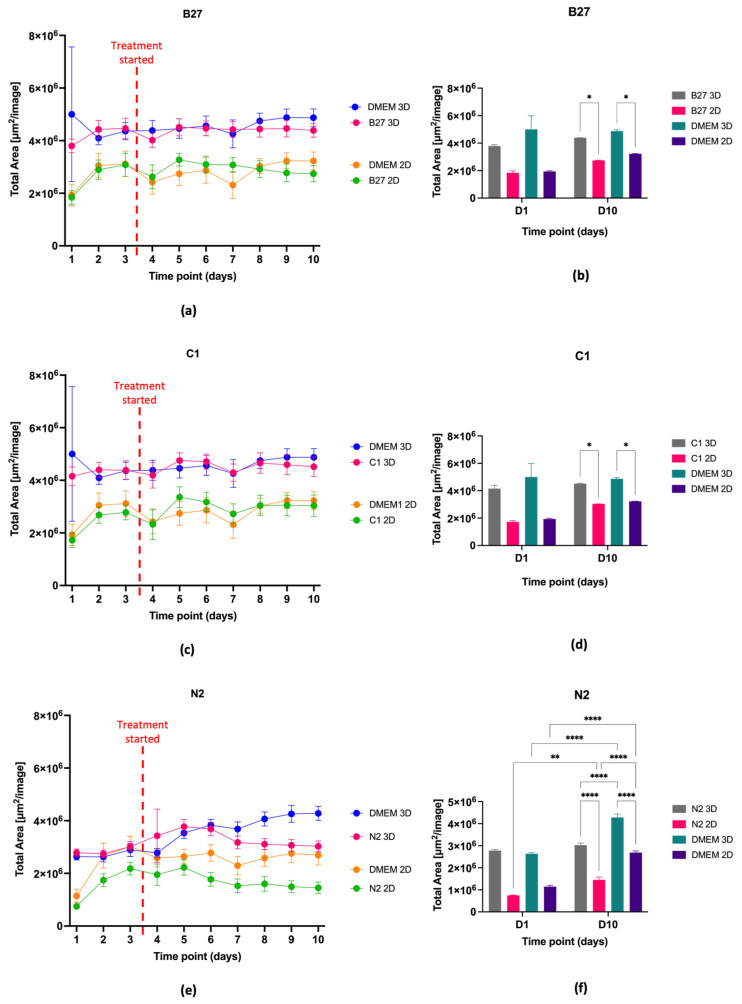
Cell confluence and proliferation over treatment course. Dot graphs show cell confluence and proliferation over time as measured by total cell area in 2D and 3D (large plug) culture for cells treated with (**a**) B27; (**c**) C1; and (**e**) N2. Bar graphs show the proliferation changes of the cells measured as total cell area comparing D1 and D10 in both 2D and 3D (large plug) for (**b**) B27; (**d**) C1; and (**f**) N2. No statistical significance *p* > 0.05; statistical significance *, *p* ≤ 0.05; statistical significance **, *p* ≤ 0.01; statistical significance ****, *p* ≤ 0.0001; interactions not shown in bar graph are not significant.

Live cell imaging analysis (Figure 2) shows that the cell confluence in the DMEM control group for both 3D and 2D steadily increases over time, while treatments cause a decrease in this variable over the treatment course (Figure 2a,c,e). The 3D samples overall show higher confluence than the 2D counterparts in all instances, with cell confluence being significantly higher in the 3D samples than in the 2D samples in all treatments (Figure 2b,d,f) (parametric two-way ANOVA with Tukey’s multiple comparisons: *p* ≤ 0.05 for B27, *p* ≤ 0.05 for C1 and ****, *p* ≤ 0.0001 for N2)

#### 2.1.2. Cell Morphology

**Figure 3:**
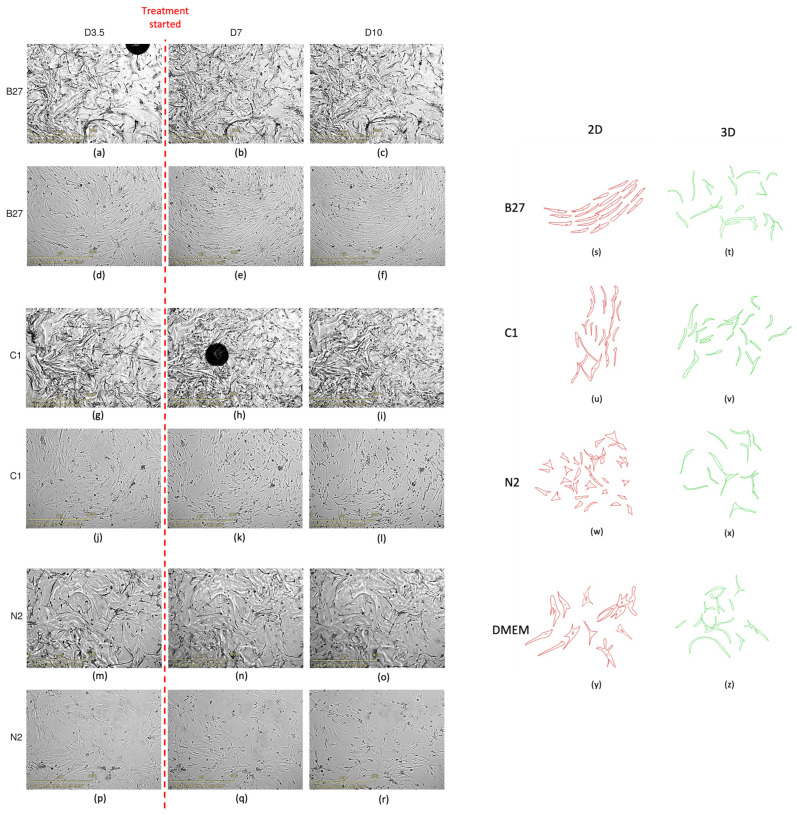
Live cell imaging over course of treatment. (**a**–**r**) Representative live cell images at time points D3.5, D7, and D10 for both 2D- and 3D-cultured cells. (**a**–**c**) B27 3D; (**d**–**f**) B27 2D; (**g**–**i**) C1 3D; (**j**–**l**) C1 2D; (**m**–**o**) N2 2D; and (**p**–**r**) N2 3D. The black round artefacts are bubbles. (**s**–**z**) Graphical representation of cell morphology changes between 2D and 3D cells in each treatment. These were manually drawn by tracing the cell shapes on a digital tablet. Scale bar in yellow is 800 μm.

Live cell imaging (Figure 3) showed changes in cell morphology and alignment in all 2D samples, with all treatments showing greater cell alignment compared to the usual disorganised arrangement of DMEM control cells. The 2D-treated cells became more polarised and aligned with one another (Figure 3d–f,j–l,p–r,s,u,w). Despite the imaging data for the 2D samples providing insight into alterations in cellular morphology, the interpretation of the images of the cells grown in 3D culture has proven challenging. No obvious morphological and cellular arrangement changes can be seen in the 3D samples (Figure 3a–c,g–i,m–o,t,v,x,z). The 3D morphology and arrangement changes are harder to visually assess, given the nature of 3D constructs and images only showing one slice of the Z plane. Therefore, morphology cannot effectively be visually assessed by imaging in the same way that morphology changes in the 2D samples can be, highlighting the need for more robust and accessible protocols.

### 2.2. Cell Characterisation

#### 2.2.1. Immunocytochemistry

**Figure 4:**
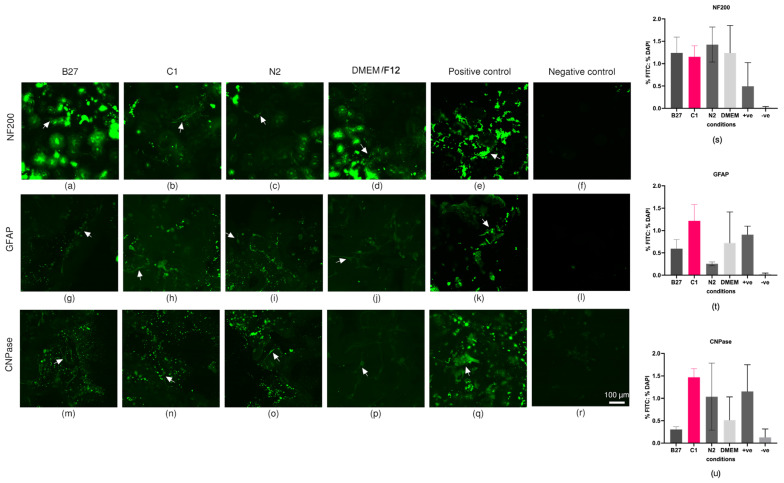
(**a**–**r**) Immunocytochemistry imaging. Representative maximum intensity projection confocal microscopy images of immunocytochemistry staining of ADSCs treated with either B27, C1, N2, or DMEM in 3D with respective positive and negative staining controls for antibody markers CNPase, NF200, and GFAP. Cells were imaged with a Nikon A1R inverted microscope using an S Plan Fluor LWD 20× 0.7NA objective. Fluorescence was captured with a laser at 488 nm excitation and GaAsP detector (500–550 nm) for AlexaFluor488-conjugated secondary antibodies (green). Scale bar = 100 μm. Note that control cells are smaller than ADSCs. Green speckles are unspecific staining and were excluded during analysis. White arrows show examples of positive cell staining. (**s**–**u**) Immunocytochemistry marker expression for 3D cells in each treatment with B27, C1, N2, and DMEM. Marker expression was measured from sum intensity projections of wide-field fluorescence images of the whole 3D plug and is displayed as the fraction of the percentage area of FITC (AlexaFluor488-conjugated secondary antibody) over the percentage area of DAPI-labelled nuclei. One-way ANOVA with multiple comparison was conducted using Bonferroni’s multiple comparison test. No statistical significance (α = 0.05) was detected.

The staining of positive control cells (Figure 4e,k,q) showed robust positivity for all antibodies and negative control cells (Figure 4f,l,r) showed no signal as expected. There were high levels of fluorescent debris in all samples stained in 3D plugs, and these were unable to be removed despite multiple washing steps. Therefore, ‘speckles’ were removed during analysis based on size and staining intensity. This is further explained in Section 4.4.1.

Immunocytochemistry analysis revealed that incubation of cells with B27, C1, and N2 supplements while suspended in PEG-based 3D matrices did not significantly increase the specific markers for neurons (NF200), oligodendrocytes (CPNase), or astrocytes (GFAP) compared to the DMEM controls. C1 was observed to have the greatest increases for CPNase and GFAP and N2 had the greatest increase in NF200 but these were not statistically significant.

#### 2.2.2. Proteomics

**Figure 5:**
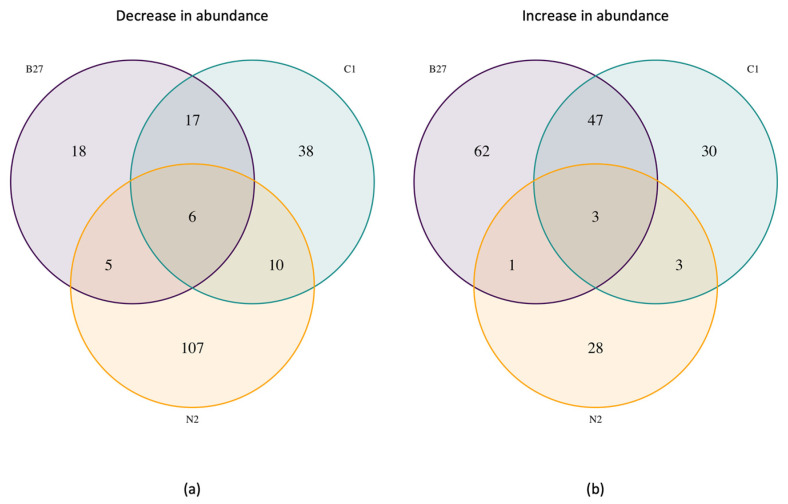
Count of proteins that have (**a**) decreased or (**b**) increased in each treatment (3D) compared to the DMEM control (3D).

Proteomics analysis revealed distinct changes between each 3D treatment and the 3D DMEM control and between the 3D treatments themselves (Figure 5). When compared to the DMEM control, 18 proteins decreased and 62 proteins increased (Log2 fold change of +/−1.5) with B27 treatment, 38 decreased and 30 increased with C1 treatment, and 107 decreased and 28 increased with N2 treatment, with only 6 decreased and 3 increased proteins common to all treatments (Figure 5).

**Figure 6:**
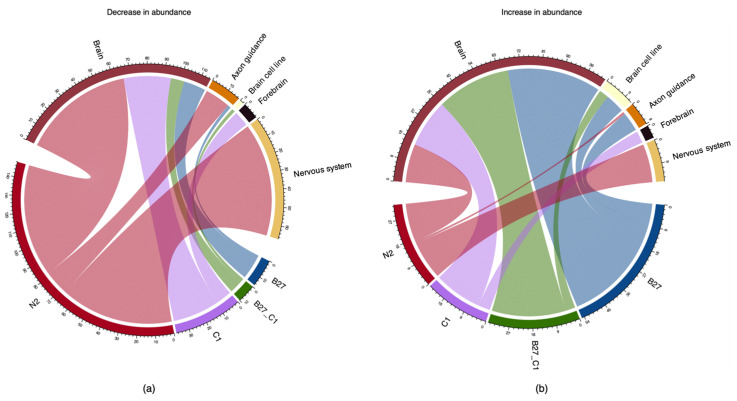
Chord diagram visualising the total number of proteins involved in different neural processes in each treatment with (**a**) being proteins with fold changes ≤ −1.5 and (**b**) being proteins with fold changes ≥ 1.5. Numbers associated with the neural function segments (top) indicate the count of proteins associated with the given neural function. Numbers associated with treatment segments (bottom) should be interpreted with caution; these indicate the count of neural functions within a given treatment (a protein corresponding to multiple neural functions will be counted multiple times accordingly).

Notably, StringDB analysis for the proteins identified for each treatment and treatment overlap revealed a range of identified proteins that were related to neural functions (Figure 6).

**Figure 7:**
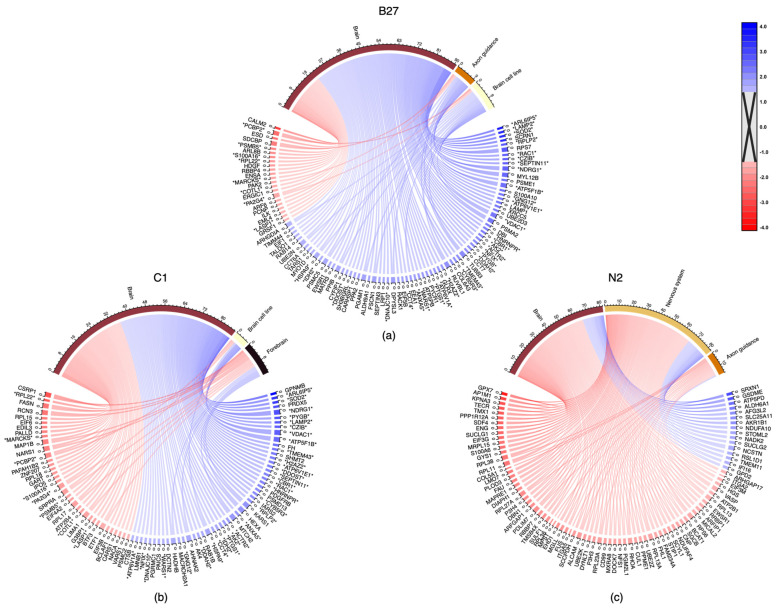
Names of proteins associated with ≥1.5-fold change or ≤−1.5-fold change (bottom) and implicated in potential neural functions by 3D treatment (top) with (**a**) B27, (**b**) C1, and (**c**) N2. Colours for a given gene indicate the relative fold change in comparison to the negative control (3D DMEM), where red indicates a negative fold change and blue indicates a positive fold change. Where a protein has a wider section allocated, this protein can be seen linked to multiple functions (e.g., CRSP1, the first protein (clockwise) in C1 treatment). * Asterisks appending and prepending protein names indicate those which that are present in both C1 and B27 (no overlap of proteins was detected among other treatment combinations).

StringDB analysis of B27-treated cells revealed 91 proteins annotated as involved in functions associated with the brain, 14 proteins involved in brain cell lines, and 10 proteins in axon guidance, with more than half of these increasing in abundance (Figure 7a). C1 treatment String analysis revealed 87 proteins annotated as involved in the brain, 6 proteins involved in brain cell lines, and 14 proteins involved in the forebrain, with more than half of these increasing in abundance (Figure 7b). N2 treatment String analysis revealed 82 proteins annotated as involved in the brain, 83 proteins involved in the nervous system, and 16 involved in axon guidance, with more than half of these decreasing in abundance (Figure 7c).

## 3. Discussion

The aim of this study was to examine the effects of the neural culture supplements B27, C1, and N2 on ADSCs grown in a 3D environment. Our previous work and the literature more broadly demonstrate that ADSCs grown in traditional 2D culture with neural culture supplements exhibit changes consistent with neural differentiation [31]. Similarly, ADSCs grown in a 3D culture system have shown a degree of differentiation toward neural cell subtypes with the expression of neural markers [30]. Thus, it was hypothesised that the supplements would further enhance neural differentiation of the ADSCs grown in 3D and there would be biological differences and changes in the expressed proteome indicative of this. The data obtained support the hypothesis that the supplements enhanced neural differentiation in the ADSCs grown in 3D compared to the untreated 3D cells. The supplements had a range of observable effects on the ADSCs with changes in proliferation, surface marker expression, and the wider proteome.

### 3.1. Cell Viability and Confluence during Supplement Treatment

Similarly, to our previous work [30], the cells grown in PEG-based 3D matrices did not experience a statistically significant decrease in viability or proliferation. In addition, treatment with either B27, C1, or N2 had no statistically significant effect on the viability and proliferation of the 3D-grown cells over the treatment course. Cell viability and confluence remained unchanged for B27- (Figure 1a,b and Figure 2a,b), C1- (Figure 1c,d and Figure 2c,d), and N2-treated (Figure 1e,f and Figure 2e,f) cells in 3D when compared to the beginning of treatment. While cell coverage fluctuated over time, there was no difference between beginning and end time points, indicating that the treatments have not compromised cell health and are not negatively impacting cell growth. It is important to note, however, that while there is no statistically significant decrease or increase in cell confluence, supplements have slowed down cell growth. It is known that as stem cells start to differentiate, proliferation decreases, and as neural cells mature, they reach the G_0_ cell cycle stage, where division stops [32]. When looking at DMEM 3D control cells, these have shown a significant increase in confluence over time (Figure 2e,f), similar to that previously reported [30]. While the treated cells’ proliferation may not have significantly decreased from when treatment started, no increase in proliferation is a sign of metabolic activity slowing down. Furthermore, the proteomics data showed that proliferating cell nuclear antigen (PCNA), a protein that regulates proliferation throughout the cell cycle [33], decreased in 3D B27 (−0.54-fold) and in 3D C1 (−0.777-fold) compared to the DMEM 3D control, supporting that the cell proliferation is slowing down in B27 and C1 compared to the DMEM 3D control.

In summary, Alamar blue and cell confluence results suggest that cell treatment has not caused significant cell loss. Still, the cell proliferation is slowing down compared to DMEM 3D control counterparts after 10 days of treatment, which may be a sign of cells starting to commit to a lineage.

### 3.2. Morphological Changes during Supplement Treatment

Cell morphology is an important aspect to consider in cell culture as it provides important information on cell changes and health [34]. Furthermore, the cell environment also plays an important role in cell functions, differentiation, migration, and morphology [35].

While no distinct morphological changes can be observed between treatments and the 3D DMEM control, cell morphology did change in 3D compared to 2D grown samples, similar to our previous work [30]. It is known that cells in 3D growth environments differ significantly morphologically and physiologically from their 2D-grown counterparts, with spatial and physical aspects of 3D cultures influencing gene expression and cellular behaviour through signal transduction from the outside in rather than inside out [22]. Two-dimensionally grown untreated ADSCs normally present an irregular fibroblastic-like morphology appearing as large, flattened cells with centrally located nuclei (Figure 3d,j,p). When these cells are placed in a PEG-based 3D matrix, their cytoskeleton rearranges [30]; cells become elongated, with a more condensed cytoplasm, lower nuclei-to-cytoplasm ratio, and spindle-like morphology. They also present clear networks and branch out; morphological features normally seen in neural cells. These morphological changes have also been observed in 2D-grown ADSCs treated with B27, C1, and N2 [31].

We previously showed that the PEG-based matrix alone started to direct cells towards a neural pathway with significant structural changes [30], suggesting that the exposure of ADSCs to supplements in the current study did not further enhance the neural morphological changes. This may be due to the timeframe used in these experiments, and more time points for 3D need to be explored. Additionally, morphological changes between treatments in 3D are likely more subtle than those observed between 2D and 3D treatments we have previously undertaken [30].

Additionally, it is important to note that 3D morphology and arrangement changes are harder to visually assess, given the nature of 3D constructs and images only showing one slice of the Z plane. Therefore, more subtle morphology changes potentially induced by the treatments cannot effectively be visually assessed by imaging in the same way that morphology changes in the 2D samples can be. Equipment limitations may also impact the ability to obtain more robust images for morphology assessment. The microscope available for high-content live cell imaging in this work is not able to perform Z-stacks, and higher-magnification images were not able to be obtained due to the working distance of the objectives and the height of the gels. This highlights the need for more robust and accessible protocols and equipment in live microscopy for 3D tissue culture.

### 3.3. Neural Surface Marker Expression Changes

The nature of the hydrogels presented difficulties in minimising non-specific staining. Despite many optimisation steps and added washing steps to the protocol, unbound antibodies are likely to be retained within the gel during staining. These presented as speckles within the images and were removed during analysis based on size and staining intensity (see Section 4.1), however, it further highlights the need for more robust staining protocols for 3D samples.

Immunocytochemistry results showed that treatment of ADSCs in PEG-based 3D matrices with B27, C1, and N2 did not significantly increase neural marker expression in the time frame they were treated.

While not statistically significant, it is important to note that C1 treatment caused the greatest increase in CNPase and GFAP marker levels that are biologically relevant and in line with that previously reported in the 2D experiments [31]. Similarly, N2 had the greatest increase in the NF200 neuronal marker and showed increased levels of CNPase and GFAP, analogous to previous 2D experiments [31], indicating that C1 cells may be differentiating towards a glial lineage. Additionally, proteomics data detected CNPase in the samples, however, the amounts detected were not significant when compared to the 3D DMEM controls.

Three-dimensional environments provide a more realistic and heterogeneous growth and unequal cell exposure to oxygen, nutrients, and treatments, creating a gradient of medium availability completely different to that in 2D environments [21,36,37]. This means that cells grown in 3D are potentially exposed to different amounts of treatments depending on their location within the gels and depending on the treatment’s ability to diffuse through the gel effectively [21,36,37]. It is likely that the effects on the cells from the treatments have been less/slower due to the nature of the 3D environment, and treatment diffusion through the matrix may mean that cells in 3D matrices require longer exposure times. It is also possible that the unequal cell exposure to the treatments may have created different cell populations at different stages of growth and differentiation. In 2D cultures, cells proliferate at similar rates, while in 3D cultures, it is normal to have a mixture of cells at different stages in the cell cycle [22] and the low numbers of markers detected may be due to only certain cell populations expressing them. In the future, single-cell proteomics methodologies will shed more light on cell-to-cell differences due to their location in the 3D environment.

Additionally, GFAP and NF200 are mature cell markers, and the ADSCs may not yet be at a differentiation stage where they are abundantly expressed and detectable by untargeted proteomics methodologies, given that neural cells take up to 108 embryonic days to differentiate into cortical neurons [38]. Nevertheless, these findings, while not statistically significant, are of biological significance in understanding how cells change and respond to treatment in 3D environments.

### 3.4. Analysis of Proteome Changes across Supplement Cells

Proteome analysis showed that all treatments caused changes in the cells, including changes in abundance of proteins annotated as being involved in neural processes (Figure 6 and Figure 7). Interestingly, most proteins involved in these processes were unique for each treatment. B27 and C1 treatments caused abundance changes in the same 39 proteins involved in neural processes by ±1.5-fold (Log2). Additionally, B27 treatment caused abundance changes in an additional 57 proteins involved in neural processes by ±1.5-fold (Log2) and C1 treatment caused ±1.5-fold (Log2) abundance changes in an additional 49 proteins involved in neural processes. N2 treatment caused changes in the abundance of 86 unique proteins involved in neural processes by at least ±1.5-fold (Log2) with no other neural-related proteins shared with any other treatment (Figure 6 and Figure 7).

#### 3.4.1. Proteome Changes Common in B27 and C1

B27 and C1 treatment proteome analysis revealed similar proteins increasing and decreasing in abundance that are annotated as being related to neural processes, suggesting changes are occurring in similar pathways.

Of particular interest is ARL6IP5 which increased in abundance with B27 by 3.71-fold (Log2) and with C1 by 3.40-fold (Log2). ARL6IP5 is a protein involved in the neurotransmitter release cycle and regulates intracellular concentrations of taurine and glutamate via SLC1A1/EAAC1 [39]. Taurine plays a role in neural development, osmoregulation, and neural protection with taurine transporters found in glutaminergic neurons [40,41,42]. Glutamate is the most abundant free amino acid in the brain and the major excitatory neurotransmitter in the central nervous system (CNS) [43], and it plays an important role in memory, neuronal development, and synaptic plasticity [44,45] Furthermore, increased expression of ARL6IP5 has been shown to induce neuronal differentiation and has been found to increase neurite length [46].

RAC1 is a neural surface antigen involved in the nerve growth factor receptor signalling pathway. It regulates many cellular responses, such as proliferation differentiation and neuronal maturation during hippocampus development [47]. It also plays a role in neuron adhesion, migration, and differentiation, dendritic spine formation, and synaptic plasticity in neurons [48] and was also found to be increased in abundance in both B27 and C1 treatments; it increased by 2.79-fold (Log2) in B27 and by 2.24-fold in C1. RAC1 is known to also play a role in regulating GABA (A) receptor synaptic stability through its role in PAK1 activation [48] and has a crucial role in dendritic growth and dendritic spine maturation through the Cdc42/Rac pathway [47,49]. Additionally, SEPTIN11, also known to be involved in neuronal cytoarchitecture for its role in dendritic arborisation and dendritic spines and GABAergic synaptic connectivity [50,51], was also increased with both B27 and C1 by 2.75-fold (Log2) and 2.25-fold (Log2), respectively. ATP6V1A protein also plays a role in neurite development and synaptic connectivity [52] and increased by 1.93-fold (Log2) with B27 and by 1.51-fold (Log2) with C1.

Lastly, NDRG1 protein, known to have a role in hormone responses, cell growth, and differentiation [53], was also found to be increased in both treatments, increasing by 2.66-fold (Log2) with B27 and by 2.94-fold (Log2) with C1. Furthermore, it has a role in cell trafficking in Schwann cells, and it is an essential protein for the maintenance and development of the peripheral nerve myelin sheath [53].

In summary, the identification of these proteins in both B27- and C1-treated cells suggests that the treatments have enhanced neural marker expression further than just the 3D environment alone in the given time frame. Furthermore, these proteins have the potential to be utilised as markers for neural differentiation of ADSCs.

#### 3.4.2. Proteome Changes Unique to B27 Treatment

In B27-treated cells, β3-tubulin (TUBB3) increased by 2.08-fold (Log2) from the 3D DMEM control. This is particularly interesting given that TUBB3 had been previously seen to increase in 3D DMEM when compared to 2D DMEM cells, showing that the environment alone increased the marker [30]. The further increase in TUBB3 observed in the 3D B27-treated cells in this study suggests that B27 treatment had a further effect on TUBB3 protein levels in the cells. β3-Tubulin is a well-known marker for early neural development; it is a major component in the neuronal cytoskeleton and is highly expressed during neural development, playing a role in maintenance, maturation, and proper axon guidance [54,55,56,57,58]. Similarly, DPYSL3, a necessary protein for signalling and cytoskeleton remodelling, was found to increase by 1.8-fold (Log2) with B27. It also plays a role in axon guidance, neuronal growth, cone collapse, and cell migration [59]. Furthermore, FSCN1 is an actin-bundling protein that is involved in the reorganisation of the actin cytoskeleton filopodia formation and axon growth cone collapse in response to nerve growth factor [60,61]. In the B27 treatment, FSCN1 increased by 1.76-fold (Log2) and its expression in the nervous system is known to increase during development and decrease after maturation [62], suggesting that the cells may be in an early neural developmental stage. CYFIP1 was also found to be increased by 1.67-fold (Log2) in B27 treatment. This particular protein plays a role in lamellipodia formation and axon outgrowth, and it is part of the WAVE complex, which regulates actin filament reorganization, also regulated by RAC1 [63] (which is also increased with B27 and C1 and addressed above). The increase in abundance of these proteins suggests that there are notable changes occurring in the cytoskeleton of the cells towards the formation of axons and that similar pathways for axon formation and cytoskeleton remodelling are being activated by B27 treatment in the ADSCs in the 3D environment.

Additionally, the increase in SEPTIN proteins in B27 treatment suggests changes in the neurodevelopment realm. SEPTIN proteins contribute to neurodevelopment and neuronal functions in the mammalian nervous system [64], with SEPTIN3 being highly expressed in the brain [65]. SEPTIN3 protein is involved in presynaptic plasticity via the cGMP/PKG pathway, and its expression is developmentally regulated as nervous system development increases [65] and it is predominantly abundant in nerve terminals and presynaptic and synaptic vesicles [65,66]. SEPTIN3 increased in abundance by 1.77-fold (Log2) with B27 together with SEPTIN11. As previously mentioned, SEPTIN11 increased with both B27 and C1, and it plays an important role in neuroarchitecture and dendrite formation [50,51]. Furthermore, LRP1 also increased by 1.79-fold (Log2) and is a membrane receptor found in neurons. It is involved in calcium signalling and neurotransmission [67] and is essential in neural development through extracellular signal transduction and intracellular signal propagation modulation [68], suggesting further development of the cytoarchitecture of neurons.

Lastly, COL6A3 (collagen VI) also increased by 2.03-fold (Log2). Collagen IV is a major protein found in the nervous system; it acts as a regulator for Schwann cell differentiation and is essential for nerve myelination preservation, function and structure, and for assisting in nerve regeneration after injury [69]. Immunocytochemistry analysis detected the CNPase marker, a well-known oligodendrocyte and myelin marker [70], suggesting the cells may start to express myelination markers.

In summary, the proteomics data for B27-treated cells suggest that B27 treatment did enhance the neural marker expression further than just the 3D environment alone in the given time frame.

#### 3.4.3. Proteome Changes Unique to C1 Treatment

In addition to the above-mentioned proteins found to be increased with both B27 and C1, C1 treatment was also associated with an increase in some proteins of interest not observed in other treatments.

PDGFRB increased in abundance by 2.18-fold (Log2) in C1-treated cells. Platelet-derived growth factors (PDGFs) and their receptors (PDGFRs) are essential proteins expressed in embryonic and mature nervous systems in neural progenitor cells, neurons, astrocytes, and oligodendrocytes [71]. They play a role in neural development and cell maintenance in the nervous system [71]. PDGF-mediated signalling has a role in regulating CNS functions such as neurogenesis, cell survival, synaptogenesis, and neuronal development [72]. Specifically, PDGFRB plays a crucial role in neuroprotection, tissue repair, and functional recovery through signalling neurons and astrocytes, as well as a role in CNS development and vascularisation [72], further supporting that the cells are committing to a neural lineage.

Additionally, DCTN2 increased in C1-treated cells by 1.61-fold (Log2), a protein that has been found to play a key role in brain development, specifically in synapse formation [73,74], further supporting changes towards synapse and dendrite formations in the cells.

In summary, the proteomics data for C1-treated cells suggest that C1 treatment did enhance the neural marker expression further than just the 3D environment alone in the given time frame. It is important to remember that C1 is a commercially available supplement designed to enhance the support of NSCs to neurons whilst minimising the persistence of progenitor cells, and when used with ADSCs it may promote the maturation of the cells.

#### 3.4.4. Proteome Changes Unique to N2

N2 proteome analysis showed a different proteome profile with no overlap with the other treatments in proteins related to neural processes, with most neural-related proteins decreasing in abundance, indicative that the treatment may not be enhancing neural development like the other treatments are. Furthermore, some of the proteins enhanced by the treatment seem to be involved in neurodegeneration. For example, GSDME was increased in abundance by 2.61-fold (Log2) and is a protein involved in mitochondrial dysfunction, axon loss, and neurite retraction and is thought to contribute to neurodegeneration [75]. Furthermore, mutations to AFG3L2 are associated with spinocerebellar ataxia involving loss of Purkinje cells [76]. Another protein which was found to increase by 2.06-fold (Log2) in N2 treatment is NCSTN, which has been linked to Alzheimer’s disease through its involvement in amyloid formation [77].

Nevertheless, it is important to note that these were short experiments with non-neuronal cells. The protein abundance profile should be further assessed for longer periods of time. Furthermore, these supplements are intended to support mature neuronal cells, and those mechanisms may be suppressing differentiation in these cells. N2, particularly, is designed for long-term support of differentiated cells, so longer time periods may be required to see more definitive changes.

In conclusion, cell proliferation, viability, immunocytochemistry, and proteomics analysis looking specifically at proteins related to neuronal processes in ADSCs treated with B27 and C1 indicates that those supplements are pushing them further down a neuronal pathway, suggesting that supplementation is a useful addition to 3D culture for the neural differentiation of ADSCs. On the other hand, however, N2 treatment did not reduce cell proliferation or show proteome changes indicative of enhanced neural marker expression similar to B27 and C1 in that short time. Ideally, these experiments should be repeated for a longer period with treatment combinations also being explored.

## 4. Materials and Methods

hADSCs from a single donor were isolated and expanded as previously described [12] with approval from the UTS Human Research Ethics Committee (ethics number 2013000437). Written informed consent was acquired for donor lipoaspirate release for research purposes only. After isolation, and prior to experiments, the cells were maintained in DMEM/F12 + Glutamax media (Gibco, Life Technologies, Carlsbad, CA, USA) with 10% heat inactivated FBS (Gibco, Life Technologies, Carlsbad, CA, USA) and incubated at 37 °C at 5% CO_2_. Cells used for these experiments were between passages five and ten.

At passage five to ten, cells were lifted from the tissue culture flasks using TrypLE express (12604 Gibco, Life Technologies, Roskilde, Denmark) and prepared for bioprinting following the manufacturer’s instructions.

Once the cells were replated in 2D or bioprinted, they were maintained in similar conditions as above for 84 h before treatment, starting with the addition of 1% antibiotics/antimycotics (ABAM, Gibco life technologies, Carlsbad, CA, USA) to maintenance media and treatments. There were a total of three experimental groups and one undifferentiated control group. The groups consisted of hADSCs treated with the treatments B27, N2, CultureOne (C1), and DMEM (undifferentiated control), respectively (Table 1). Control cells were also included. The experimental design is outlined in more depth in Table 1. Media were changed every 84 h following the experimental design from Table 1 and incubated at 37 °C at 5% CO_2_. The 2D cells were added as a viability control.

### 4.1. The 3D Bioprinting of hADSCs in PEG-Based Hydrogels

hADSCs were 3D printed in a PEG-based hydrogel using a RASTRUM bioprinter (Inventia, Sydney, Australia). The cell plugs were printed into 96-well plates following the manufacturer’s instructions using the same protocol and matrices as previously described in Pelegri et al., 2023, with 24 plugs printed for each treatment for downstream analysis [30].

In brief, a large plug and imaging plug were printed at ~1.1 kPa containing RGD and YIGSR peptides (matrix code PX02.21P). RGD and YIGSR were included in the system given that the peptide trimer RGD is found in collagen, laminin, and fibronectin, which mediates the adhesion of many cells, including neurons [78], and laminin-derived (YIGSR) peptide is known to promote neuronal cell binding [79].

**Figure 8:**
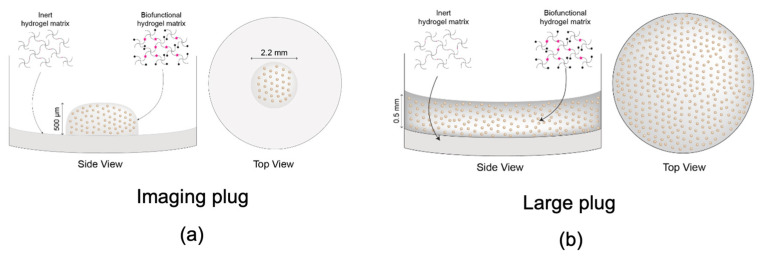
Visual representation and dimensions of the different constructs provided by Rastrum. (**a**) Smaller plug, referred to as imaging plug used for immunocytochemistry; (**b**) Larger plug, referred to as large plug (used for viability assays, live cell imaging, and proteomics. Graphics reproduced from Pelegri et al., 2023.

Cells were seeded at a concentration of 10 million/mL. The imaging plug consisted of a small volume of hydrogel with embedded cells in the centre of the well measuring 0.5 mm in height and 2.2 mm in diameter (Figure 8a). The large plug occupied the well completely, measuring 0.5 mm in height and 5 mm in diameter (Figure 8b). The 2D-seeded cells were also included as morphology and viability controls. Cells were lifted from the tissue culture flasks using TrypLE express (12604 Gibco, Life Technologies, Roskilde, Denmark) and replated into 96-well plates in 2D conditions at 10 million/mL, the same concentration as 3D cells. These were grown in parallel and treated the same way. The only difference was the 3D construct vs. 2D environment.

Positive controls for immunocytochemistry were included and printed in parallel to the ADSCs following the same method. These are further explained in Immunocytochemistry Section 4.4.1.

### 4.2. Cell Morphology: Incucyte Imaging

Live images were taken every 24 h using the organoid programme in the Incucyte^®^ S3 Live-Cell Analysis Instrument (Sartorius, Göttingen, Germany) for 10 days following the same methods as Pelegri et al., 2023 [30].

In brief, cell confluence was assessed as the area covered by cells in each image (um^2^/image). This was conducted using the instrument’s inbuilt analysis software. Parameters were set to: radius 200; sensitivity 70; edge sensitivity 0; hole fill (um²) 500; adjust size (pixels) 0. After the initial analysis was finalised by the instrument, all images at all time points were manually checked for artifacts that would not accurately represent the confluence. Common artifacts found in the images were glares and bubbles which prevented the camera from taking an accurate photo of the cell coverage. Further analysis and graphing were performed using the data exported from the Incucyte^®^ proprietary software (version 2022B Rev2). Averages of total area for wells of each cell type and per time point, with associated standard deviations for both for 2D and 3D models, were plotted. The dataset was assessed for normality using Shapiro–Wilk test and statistical significance was subsequently determined using parametric two-way ANOVA with Tukey’s multiple comparisons. GraphPad PRISM software (version 10.0.3) was used for data visualisation (Figure 3).

### 4.3. Cell Viability and Proliferation: Alamar Blue

Cell viability assay was performed at 3 different time points: D3.5, D7, and D10.5, using an Alamar blue assay. Alamar blue is a non-toxic cell viability assay that detects metabolically active cells. When Alamar blue is added to cells, if cells are metabolically active, the main active ingredient, resazurin, is reduced to resorufin, and the solution becomes red in colour and highly fluorescent.

Alamar blue (10% in media) was added to the cells and left to incubate for 16 h to allow enough time to penetrate through the 3D matrices. To keep variables to a minimum, the same was carried out on the 2D cells. Negative control wells were included; these only contained the Alamar blue and clean media mixture. After the incubation period, the Alamar blue and media mixture was transferred to a different 96-well plate to keep the cellular growth environment as undisturbed as possible from outside factors. The collected Alamar blue media were measured using the fluorescence bottom-up mode in a Tecan M200Plate Reader (Tecan, Männedorf, Switzerland) using 530–560 nm excitation and 590 nm emission wavelengths. The results were averaged across the 96 wells, and data were normalised to the negative controls. The data were analysed as fold change ratio values from D3.5. The dataset was assessed for normality using the Shapiro–Wilk test, and due to the assumption not being met, a non-parametric Kruskal–Wallis test was performed to determine significance. GraphPad PRISM software was used for data visualisation (Figure 1).

### 4.4. Cell Characterisation

#### 4.4.1. Immunocytochemistry

Cells from the imaging plug were fixed using 10% formalin for 30 min prior to washing and storing in PBST + 0.1% *w*/*v* sodium azide at 4 °C. For staining, cells were first placed in PBST (0.01 M PBS and 0.1% Triton X-100 (BDH #30632) at pH 7.4) for 1 h at room temperature. Primary antibodies were diluted in PBG (0.1 M PBS, pH 7.4, 0.1% Triton-X, 2% NGS, 1% BSA (Sigma Aldrich, St. Louis, MO, USA, #A9647)) and were added to the relevant wells and incubated at 4 °C for 3 days. Primary antibodies were rabbit anti-glial fibrillary acidic protein (GFAP) (1/500, Dako, Glostrup, Denmark #Z0334) as an astrocyte marker; mouse anti-2′,3′ cyclic-nucleotide 3′ phosphodiesterase (CNPase) (1/100 Abcam, Cambridge, UK #ab6319-100) as an oligodendrocyte marker; and mouse anti-neurofilament 200 (1/50, biosensis CM998100) as a mature neuron marker.

After primary antibody incubation was completed, cells were then washed with three changes of PBST for 30 min and incubated with goat anti-mouse AF488 (1/200, Invitrogen, Carlsbad, CA, USA #A11001) or goat anti-rabbit AF488 (1/200, Invitrogen, #A11008) secondary antibodies in PBG for another 3 days at 4 °C. Following an additional two 20 min washes with PBST, cells were incubated with Hoechst (1/5000 Invitrogen) for 30 min to stain the nuclei and finally washed three times with PBST for another 30 min each and stored in antifade/glycerol at 4 °C until imaged.

Positive staining control cells at 10 million/mL conc were included in all staining runs. Glioblastoma U87MG cells were used for GFAP and CNPase positive staining controls. Neuroblastoma SHSY-5Y cells were used for NF200 positive staining controls. Both U87MG and SHSY-5Y cells were grown in separate plates to the experimental cells; however, the cells were grown and stained in parallel with the experimental plates for each antibody and were fixed and stained following the same protocol as the experimental cells. U87MG and SHSY-5Y cells were grown in a 96-well plate with DMEM/F12 + Glutamax media (Gibco, Baltimore, MA, USA) enriched with 10% heat-inactivated FBS (Sigma Aldrich) until confluent.

Wide-field fluorescence microscopy was performed using a Nikon Ti inverted microscope with a ×10 0.3 numerical aperture Plan Fluor objective, NIS Elements acquisition software (version 5.30.06) with a solid state Lumencor illumination source, and a Nikon DS-Qi2 CMOS camera. Six 1024 × 1024 fields of view were captured, covering the area of each imaging plug and stitched using the NIS Elements acquisition software with default overlap settings. Series of images were captured through the z dimension using a step size of 5.6 µm.

Wide-field fluorescence images were processed using the Clarify.ai algorithm using the NIS Elements acquisition software. FIJI (FIJI is just ImageJ) version 1.53 t [108] was used for image analysis. Where appropriate, Z-stacks were corrected for axial drift using the Linear Registration with SIFT plugin with an expected translation transformation. Sum intensity projections for FITC and DAPI channels were subjected to background subtraction with a rolling ball of 50 pixels, before being thresholded using the Li (FITC) or Triangle (DAPI) algorithm to create a binary mask and area fraction was measured. To eliminate non-specific secondary antibody aggregates from measurements, only particles with a pixel size larger than 100 pixels2 and a circularity of 0–0.8 were quantified. Marker expression was measured from sum intensity projections of wide-field fluorescence images of the whole 3D plug and is displayed as the fraction of the percentage area of FITC (AlexaFluor488-conjugated secondary antibody) over the percentage area of DAPI-labelled nuclei. One-way ANOVA with multiple comparisons was conducted using Bonferroni’s multiple comparison test. No statistical significance *p* > 0.05; statistical significance *, *p* ≤ 0.05; statistical significance **, *p* ≤ 0.01; statistical significance ***, *p* ≤ 0.001; statistical significance ****, *p* ≤ 0.0001.

Representative images were captured using a Nikon A1R inverted confocal microscope (Nikon, Tokyo, Japan) with a ×20 0.7 numerical aperture LWD S Plan Fluor objective and NIS Elements acquisition software. AF488 was imaged with an excitation of 488 nm, and emission detected with a GaAsP detector at 500–550 nm. FITC was captured with a 488 nm laser intensity of 3.5, gain of 40, and offset of—3. Z-stacks were acquired with a step size of 3 µm. Fluorescence images were processed using the denoise.ai algorithm using the NIS Elements acquisition software, and maximum intensity projections were created. In Figure 4, the displayed dynamic range for FITC for 3D samples is 0–175, whilst for positive controls, the displayed dynamic range for FITC is 0–2000.

#### 4.4.2. Proteomics

##### Protein Extraction

Cells were released from the 3D large plugs using the Rastrum cell retrieval protocol provided by the company. In brief, media from printed 3D cell models were discarded, cells were washed with PBS, and cell retrieval solution was added to the wells. The wells were then incubated at 37 °C for 30 min. Cells were then collected by pipetting up and down in each well and then were transferred to the collection tubes. The wells were then further washed with PBS and the remaining cells were combined in the tubes. Cells were then centrifuged and supernatant was removed. The cell pellets were then frozen until ready to be used for proteomics. Six wells were pooled to make one proteomics sample.

Once ready, samples were defrosted and resuspended in 1% SDC, 5 mM TCEP, 10 mM IAA, 100 mM HEPES pH 8.5, heated to 95 °C for 5 min, and incubated for an hour at room temperature. After the incubation, 0.1 ug of trypsin was added to 10 ug of sample and incubated at 37 °C overnight. The peptides were then recovered using the SDB-RPS-based stage tip column method, which is a modified protocol from Rappsilber et al. (2007). The cell digests were centrifuged at maximum speed for 5 min to digest any insoluble material. Then, 10× the volume of digest was added of SPE load buffer (90% acetonitrile, 1% trifluoroacetic acid). The sample was mixed by trituation and was then added to the stage tip column which contained 1 disc of SDB-RPS cut with an 18-gauge blunt-end needle. The liquid was centrifuged through the disc at 5000 rpm until all liquid moved through. Following this, two washing steps were performed to help wash any contaminants and salts from the column and bound peptides. Firstly, 100 μL of SPE load buffer was passed through at 5000 rpm until all liquid moved through followed by a second wash using 100 μL of SPE wash buffer (10% acetonitrile, 0.1% trifluoroacetic acid). After, the peptides were eluted directly into the injection vials by washing the column with 50 μL of SPE elution buffer (71 μL of 1 M ammonia solution, 800 μL of 100% acetonitrile, 129 μL of water) and centrifuging at 5000 rpm until all liquid passed through the column into the vials. The vials containing the peptides were then placed into a vacuum centrifuge (Savant DNA 120, SpeedVac Concentrator, Thermo Scientific, Carlsbad, CA, USA) to evaporate all liquid. Once samples were dry, the peptides were resuspended using 25 μL of MS loading solvent (2% acetonitrile, 0.2% trifluoroacetic acid) and samples were ready to be analysed by LC-MS/MS.

##### LC-MS/MS Analysis

Using an Acquity M-class nanoLC system (Waters, Milford, MA, USA), 5 µL of the sample was loaded at 15 μL/min for 3 min onto a nanoEase Symmetry C18 trapping column (180 μm × 20 mm) before being washed onto a PicoFrit column (75 μm × 350 mm; New Objective, Woburn, MA, USA) packed with SP-120-1.7-ODS-BIO resin (1.7 μm, Osaka Soda Co., Osaka, Japan) heated to 45 C at 300 nL/min. Peptides were eluted from the column and into the source of a Q Exactive Plus mass spectrometer (Thermo Scientific) using the following program: 5–30% MS buffer B (98% acetonitrile + 0.2% formic acid) over 90 min, 30–80% MS buffer B over 3 min, 80% MS buffer B for 2 min, 80–85% for 3 min. The eluting peptides were ionised at 2400 V. A data-dependent MS/MS (dd-MS2) experiment was performed, with a survey scan of 350–1500 Da performed at 70,000 resolution for peptides of charge state of 2+ or higher with an AGC target of 3 × 10^6^ and maximum injection time of 50 ms. The top 12 peptides were selected and fragmented in the HCD cell using an isolation window of 1.4 *m*/*z*, an AGC target of 1 × 10^5^, and maximum injection time of 100 ms. Fragments were scanned in the Orbitrap analyser at 17,500 resolution and the product ion fragment masses measured over a mass range of 120–2000 Da. The mass of the precursor peptide was then excluded for 30 s.

##### Data Processing and Analysis

Raw files from the Q Exactive Plus were searched using MaxQuant (version 2.0.3.0) hosted on the Galaxy Australia platform against the UniProt *Homo sapiens* database (downloaded on 23 March 2023) using the following specific parameters settings. Min. peptide length: 7. Max. peptide mass (Da): 4600. Min. unique peptides: 0. Calculate peak properties: false. Match between runs: true. Match time window (min): 0.7. Match ion mobility window: 0.05. Alignment time window (min): 20. Alignment ion mobility: 1. Match unidentified features: false. Include contaminants: true. Decoy mode: revert. PSM FDR: 0.01. Protein FDR: 0.01. Min. peptide length for unspecific searches: 8. Max. peptide length for unspecific searches: 25. Peptides for quantification: unique + razor. Use only unmodified peptides: true. Separate LFQ in parameter groups: false. Stabilize large LFQ ratios: true. Require MS/MS for LFQ comparisons: true. Missed cleavages: 2. Fixed modifications: nothing selected. Variable modifications: oxidation (M) carbamidomethyl (C) deamination (NQ). Enzyme: trypsin/P. Digestion mode: semi-specific. Quantitation methods: LFQ. LFQ min. ratio count: 2. LFQ min. number of neighbours: 3. LFQ average number of neighbours: 6. Normalization type: classic.

The Protein Groups file from the MaxQuant search was then input in LFQ Analyst (Dev.) (https://bioinformatics.erc.monash.edu/apps/LFQ-Analyst/; accessed on 1 September 2023 [80]) for further analysis. LFQ analyst was set to a 0.05 *p*-value cutoff, 1.5 Log2 fold change cutoff with Perseus-type imputation, no normalization, and Benjamini–Hochberg FDR correction.

StringDB analysis was conducted using String V.11 using the following analysis parameters: Network type: full string network; meaning of network edges: evidence; active interaction sources: textmining, experiments, databases, co-expression, neighbourhood, gene fusion, co-occurrence; minimum required interaction score: medium confidence (0.400); max number of interactors to show: 1st shell—non/query proteins only. 2nd shell—none; network display mode: interactive SVG; network display options: disable 3D bubble design, disable structure previews inside network bubbles. Scripts used for data processing and visualisation of proteomic data are available on GitHub (https://github.com/maxlcummins/Pelegri_et_al_2023; accessed on 6 October 2023).

## Figures and Tables

**Table 1:** Outline of experimental treatments and cell types.

Treatment	Cell Type	Base Media	Supplement
B27	hADSC	Neurobasal media	1% B27 supplement
CultureOne	hADSC	Neurobasal media	1% CultureOne (C1)
N2	hADSC	Neurobasal media	1% N2 supplement
DMEM (undifferentiated control)	hADSC	DMEM/F12 + Glutamax—control media	10% FBS
Positive Staining controls	SHSY-5Y or U87MG	DMEM/F12 + Glutamax—control media	10% FBS
Negative staining controls	hADSC	DMEM/F12 + Glutamax—control media	10% FBS

## Data Availability

Proteomics LFQ analyst data, String data, and scripts used for data processing and visualization of the proteomics experiments are available on GitHub (https://github.com/maxlcummins/Pelegri_et_al_2023; accessed on 6 October 2023).
